# Supplementary material for: Metabolic Syndrome and Dietary Habits in Hospitalized Patients with Schizophrenia: A Cross-Sectional Study
Source: Medicina (Kaunas). 2021 Mar 10;57(3):255. doi: 10.3390/medicina57030255 (PMC8001284; doi:10.3390/medicina57030255)
Supplement: Supplementary file 1 [file medicina-57-00255-s001.pdf]

SUPPLEMENTARY MATERIAL

Supplementary Table S1. Anthropometric characteristics, blood pressure, and biochemical parameters of the study participants.

Parameter	with MetS (*n* = 124) Mean ± SD (95% CI)	without MetS (*n* = 135) Mean ± SD (95% CI)	*p*[c]
BW (kg)	90.36 ± 16.51 (87.43–93.30)	75.42 ± 14.71 (72.92–77.93)	<0.001
BH (cm)	176.62 ± 8.97 (175.03–178.22)	177.59 ± 8.71 (176.08–179.04)	0.395
BMI (kg/m^2)	28.97 ± 4.96 (28.08–29.85)	23.90 ± 4.29 (23.17–24.63)	<0.001
WC (cm)	108.75 ± 11.57 (106.69–110.81)	94.35 ± 12.14 (92.28–96.41)	<0.001
HC (cm)	107.87 ± 9.49 (106.18–109.55)	100.06 ± 8.47 (98.62–101.50)	<0.001
WHR	1.01 ± 0.06 (1.00–1.02)	0.94 ± 0.08 (0.93–0.95)	<0.001
BF [a] (%)	29.12 ± 8.69 (27.53–30.71)	20.52 ± 9.05 (18.85–22.20)	<0.001
BF [a] (kg)	27.08 ± 11.02 (25.07–29.10)	16.40 ± 8.98 (14.74–18.06)	<0.001
SBP (mmHg)	132.36 ± 19.57 (128.88–135.84)	120.77 ± 14.26 (118.34–123.19)	<0.001
DBP (mmHg)	83.53 ± 9.07 (81.92–85.14)	78.91 ± 7.78 (77.58–80.23)	<0.001
TC (mmol/L)	4.98 ± 1.21 (4.77–5.20)	4.61 ± 1.07 (4.43–4.79)	0.009
LDL-C [b] (mmol/L)	3.12 ± 1.02 (2.93–3.30)	2.86 ± 0.97 (2.69–3.02)	0.039
HDL-C (mmol/L)	0.95 ± 0.28 (0.90–1.00)	1.22 ± 0.31 (1.17–1.27)	<0.001
TG (mmol/L)	2.05 ± 1.03 (1.87–2.23)	1.17 ± 0.44 (1.09–1.24)	<0.001
GLC (mmol/L)	6.17 ± 1.84 (5.84–6.49)	4.97 ± 0.60 (4.87–5.07)	<0.001

MetS, metabolic syndrome; n, number of participants; SD, standard deviation; CI, confidence interval; BW, body weight; BH, body height; BMI, body mass index; WC, waist circumference; HC, hip circumference; WHR, waist-to-hip ratio; BF, body fat; SBP, systolic blood pressure; DBP, diastolic blood pressure; TC, total cholesterol; LDL-C, low-density lipoprotein cholesterol; HDL-C, high-density lipoprotein cholesterol; TG, triglycerides; GLC, glucose; [a] data missing for *n* = 7 with MetS and *n* = 20 without MetS (due to tremor); [b] data missing for *n* = 4 with MetS (due to the fact that TG levels were ≥ 4.52 mmol/L); [c] independent samples t-test. Statistically significant: $p < 0.05$.

Supplementary Table S2: Frequency of answers to individual questions that formed part of a non-quantitative food frequency questionnaire for participants with and without metabolic syndrome – questions related to the intake of food items/groups.

Parameter	with MetS (*n* = 124) *n* (%)				without MetS (*n* = 135) *n* (%)			
	**every day**	**few times a week**	**rarely**	**never**	**every day**	**few times a week**	**rarely**	**never**
bread and bagels: wheat or mixed wheat flour	101 (81.5)	17 (13.7)	6 (4.8)	0 (0.0)	107 (79.3)	23 (17.0)	3 (2.2)	2 (1.5)
bread and bagels: rye or whole-wheat flour	5 (4.0)	13 (10.5)	16 (12.9)	90 (72.6)	1 (0.7)	11 (8.1)	14 (10.4)	109 (80.7)
burek, puff-pastry, donuts, strudels, and similar bakery products	7 (5.6)	44 (35.5)	52 (41.9)	21 (16.9)	13 (9.6)	43 (31.9)	42 (31.1)	37 (27.4)
breakfast cereals, muesli	1 (0.8)	18 (14.5)	77 (62.1)	28 (22.6)	2 (1.5)	18 (13.3)	79 (58.5)	36 (26.7)
cakes	4 (3.2)	34 (27.4)	64 (51.6)	22 (17.7)	5 (3.7)	33 (24.4)	77 (57.0)	20 (14.8)
butter, margarine	30 (24.2)	71 (57.3)	17 (13.7)	6 (4.8)	23 (17.0)	74 (54.8)	33 (24.4)	5 (3.7)
eggs	2 (1.6)	75 (60.5)	36 (29.0)	11 (8.9)	4 (3.0)	96 (71.1)	25 (18.5)	10 (7.4)
jam, honey	20 (16.1)	61 (49.2)	27 (21.8)	16 (12.9)	24 (17.8)	79 (58.5)	27 (20.0)	5 (3.7)
cured meat products (sausages, hot-dogs, salami, prosciutto, budjola)	16 (12.9)	70 (56.5)	32 (25.8)	6 (4.8)	12 (8.9)	79 (58.5)	28 (20.7)	16 (11.9)
low-fat milk and dairy products	34 (27.4)	41 (33.1)	14 (11.3)	35 (28.2)	19 (14.1)	56 (41.5)	19 (14.1)	41 (30.4)
whole milk and dairy products	24 (19.4)	62 (50.0)	25 (20.2)	13 (10.5)	23 (17.0)	68 (50.4)	29 (21.5)	15 (11.1)
semi-hard cheese (e.g. Emmental cheese)	9 (7.3)	65 (52.4)	44 (35.5)	6 (4.8)	9 (6.7)	75 (55.6)	34 (25.2)	17 (12.6)
fruits	16 (12.9)	66 (53.2)	39 (31.5)	3 (2.4)	14 (10.4)	76 (56.3)	36 (26.7)	9 (6.7)

chocolate, candies, cookies, pudding	24 (19.4)	46 (37.1)	42 (33.9)	12 (9.7)	21 (15.6)	45 (33.3)	53 (39.3)	16 (11.9)
nuts (walnuts, hazelnuts, almonds)	1 (0.8)	5 (4.0)	27 (21.8)	91 (73.4)	3 (2.2)	1 (0.7)	25 (18.5)	106 (78.5)
snacks (salted sticks, potato chips, flips)	5 (4.0)	31 (25.0)	48 (38.7)	40 (32.3)	6 (4.4)	33 (24.4)	42 (31.1)	54 (40.0)
potato	16 (12.9)	104 (83.9)	3 (2.4)	1 (0.8)	11 (8.1)	116 (85.9)	7 (5.2)	1 (0.7)
pasta	1 (0.8)	111 (89.5)	10 (8.1)	2 (1.6)	2 (1.5)	118 (87.4)	12 (8.9)	3 (2.2)
rice	0 (0.0)	104 (83.9)	17 (13.7)	3 (2.4)	0 (0.0)	116 (85.9)	17 (12.6)	2 (1.5)
vegetables	35 (28.2)	72 (58.1)	16 (12.9)	1 (0.8)	41 (30.4)	77 (57.0)	14 (10.4)	3 (2.2)
red meat (e.g. beef, pork)	2 (1.6)	100 (80.6)	18 (14.5)	4 (3.2)	0 (0.0)	110 (81.5)	17 (12.6)	8 (5.9)
poultry (chicken, turkey)	0 (0.0)	111 (89.5)	12 (9.7)	1 (0.8)	2 (1.5)	115 (85.2)	11 (8.1)	7 (5.2)
fish (including clams and mollusks)	0 (0.0)	15 (12.1)	97 (78.2)	12 (9.7)	0 (0.0)	7 (5.2)	113 (83.7)	15 (11.1)
fast food (e.g. burgers, French fries)	5 (4.0)	16 (12.9)	28 (22.6)	75 (60.5)	6 (4.4)	9 (6.7)	29 (21.5)	91 (67.4)
semi-prepared and prepared food (instant, deep frozen, canned)	2 (1.6)	72 (58.1)	31 (25.0)	19 (15.3)	1 (0.8)	69 (51.1)	37 (27.4)	28 (20.7)

MetS, metabolic syndrome; n, number of participants.

Supplementary Table S3: Frequency of answers to individual questions that formed part of a non-quantitative food frequency questionnaire for participants with and without metabolic syndrome – questions related to the intake of beverage items/groups.

Parameter	with MetS (*n* = 124) *n* (%)				without MetS (*n* = 135) *n* (%)			
	**several times a day**	**once a day**	**rarely**	**never**	**several times a day**	**once a day**	**rarely**	**never**
coffee with caffeine, black tea	64 (51.6)	32 (25.8)	14 (11.3)	14 (11.3)	61 (45.2)	33 (24.4)	26 (19.3)	15 (11.1)
fruit juice, lemonade	19 (15.3)	41 (33.1)	52 (41.9)	12 (9.7)	14 (10.4)	44 (32.6)	53 (39.3)	24 (17.8)
herbal tea, fruit tea	59 (47.6)	30 (24.2)	24 (19.4)	11 (8.9)	61 (45.2)	36 (26.7)	29 (21.5)	9 (6.7)
hot cocoa, hot chocolate	2 (1.6)	13 (10.5)	74 (59.7)	35 (28.2)	2 (1.5)	14 (10.4)	93 (68.9)	26 (19.3)
carbonated soft drinks	33 (26.6)	21 (16.9)	39 (31.5)	31 (25.0)	31 (23.0)	26 (19.3)	52 (38.5)	26 (19.3)
light drinks (with reduced energy value)	3 (2.4)	4 (3.2)	9 (7.3)	108 (87.1)	0 (0.0)	2 (1.5)	12 (8.9)	121 (89.6)
carbonated mineral water	8 (6.5)	15 (12.1)	44 (35.5)	57 (46.0)	6 (4.4)	17 (12.6)	45 (33.3)	67 (49.6)

MetS, metabolic syndrome; n, number of participants.
